# Smombie Guardian: We watch for potential obstacles while you are walking and conducting smartphone activities

**DOI:** 10.1371/journal.pone.0197050

**Published:** 2018-06-26

**Authors:** Donghee Kim, Kyungsik Han, Jeong Seop Sim, Youngtae Noh

**Affiliations:** 1 Department of Computer Engineering, Inha University, Incheon, Republic of Korea; 2 Department of Software and Computer Engineering, Ajou University, Suwon, Republic of Korea; 3 Department of Data Science, Ajou University, Suwon, Republic of Korea; Beihang University, CHINA

## Abstract

With the growing dependence on smartphones for everyday activities, a large number of pedestrians nowadays are constantly fixated on their smartphone screens, and hence are susceptible to walking off pavements or colliding with other pedestrians. Reduced attention and situational awareness can render smartphone-occupied users, or smombies, oblivious to potential risks when using their smartphones while walking or driving. In this paper, we introduce a smartphone application, called Smombie Guardian, that detects obstacles and alerts smombies as they walk while viewing their smartphone screens to prevent potential collisions. Based on a user study with 74 participants who used Smombie Guardian in a real-life scenario, we highlight the effectiveness, usefulness, and unobtrusiveness of the algorithm and Smombie Guardian in helping users to avoid potential obstacles.

## Introduction

The global rate of smartphone usage has continued to increase significantly in recent years, such that they now constitute a major part of most people's daily lives. According to a Pew Research report, smartphone ownership rates have skyrocketed in many countries since 2013, when the global median of smartphone ownership rate was 43%. Smartphone ownership in the US was at 72%, whereas South Korea stood out as the country with the highest smartphone ownership rate (88%) [1]. Moreover, a report from Statista has forecast that the number of smartphone users worldwide will reach 2.8 billion by 2020 (https://bit.ly/2dk8wHh). The proliferation of smartphones, and their connectivity and computational capabilities, have empowered people to engage in the sharing and acquisition of various types of information, anytime and anywhere, which goes beyond merely making and receiving phone calls.

However, owing to people's excessive reliance on smartphones and the variety of contents accessible through them, these devices tend to garner a considerable amount of user attention in many cases. At times, this absorption in smartphone screens renders people oblivious to their surroundings [2]. As reduced situational awareness causes pedestrians, in particular, to become inattentive to hazards in their environments, occurrences of accidents and injuries pertaining to such oversights are not uncommon. Such pedestrians are now referred to as smartphone zombies (henceforth *smombies*).

The number of accidents involving smombies is reportedly on the rise [2]. Based on the National Electronic Injury Surveillance System (NEISS) database, Nasar *et al*. [3] reported that the number of pedestrian injuries due to mobile phone usage increased from 559 in 2004 to 1,506 in 2010. Furthermore, the authors reported that the number of accidents occurring due to pedestrians' use of smartphones was higher than that of driving accidents in 2011. A survey by the University of Washington found that nearly one third of Americans operate their smartphones at dangerous intersections [4]. Big cities have more smombies on the streets, subways, shopping malls, and corridors of buildings. The Department of Transportation has reported that 25% of fatal accidents on urban roads in 2014 involved a pedestrian who had failed to look properly, whereas the number of accidents caused by drivers and pedestrians failing to observe their surroundings has risen by 12% over the past decade. This establishes a clear connection between such habits and an increase in accidents involving pedestrians [5]. Because people's dependence on smartphones is clear, and as a number of games and applications cause users to become highly engaged and easily distracted (e.g., Pokemon Go, it is crucial to handle potential collisions in advance and guarantee the safety of both smartphone users (smombies) and other pedestrians or drivers.

In the existing literature, many solutions have been proposed and implemented to ensure pedestrian safety against the distractions caused by immersion in smartphones. In this paper, to better ensure the safety of smombies and people in their vicinity, we introduce a smartphone app called Smombie Guardian to provide distracted smartphone users (i.e., smombies) with timely alerts to prevent potential collisions.

Smombie Guardian represents a major departure from existing work in two aspects:

- We prototyped Smombie Guardian to accurately detect the size of and distance to an impending obstacle by tracking the ratio of the user's displacement to the variation in image size. The app warns users of unsafe situations (e.g., colliding with obstacles or other pedestrians) at a distance of three meters from an obstacle (this default can be altered by users) by triggering suitable alerts in a timely manner.
- Our app reflects an empirical understanding of user experience and perception, based on a user study using A/B testing (experiments with and without Smombie Guardian) involving 74 participants. The results highlighted participants' highly positive experiences, as well as their reactions to the usefulness and efficiency of Smombie Guardian. The participants also suggested design implications for future apps of this kind, such as the ability to customize the type and frequency of alerts and detect potential obstacles in other directions.

## Related work

### Smartphone applications and technology for pedestrian safety

Wang *et al*. [6] proposed WalkSafe, a smartphone app for enhancing the safety of people crossing roads while talking on their phones. WalkSafe uses the rear camera of the smartphone to detect approaching vehicles via machine learning algorithms implemented on the phone. Jain *et al*. [7] recently proposed a pedestrian safety app called LookUp, which uses shoe-mounted inertial sensors to profile ground gradients and step patterns in order to detect sidewalk-street transitions (i.e., stepping over a curb or walking down sidewalk ramps). They reported that the detection rates of LookUp were over 90%, with 0.7% false positives. However, WalkSafe appears to improve smartphone users' safety only while they walk and talk, and LookUp requires shoe-mounted inertial sensors. Foerster *et al*. [8] proposed SpareEye, an Android app that warns pedestrian users concerning activities that require continuous focus on the screen (e.g., texting, watching videos, and gaming). SpareEye only requires the built-in camera of the smartphone. However, SpareEye relies on variations in image sizes obtained by a monocular camera, and hence cannot accurately measure the sizes of obstacles and the distances between users and obstacles. However, Smombie Guardian accurately computes the sizes of obstacles and the distances to them in a novel manner, by tracking the ratio of the user's displacement to the variation in image size (see Implementation).

### Pedestrian and obstacle detection

Pedestrian and obstacle detection are rapidly evolving areas in computer vision, and have key applications in intelligent vehicles, surveillance, advanced robotics, the assistance of visually impaired people, and so on.

For the detection of pedestrians, detectors typically follow a sliding window paradigm that entails feature extraction, binary classification, and dense multi-scale scanning of detection windows, followed by non-maximum suppression (NMS). Dollar *et al*. [9] recently evaluated 16 pre-trained state-of-the-art detectors (VJ, Shapelet, HOG, and others) across six datasets (i.e., Caltech, ETH, Caltech-JAPAN, TUD-Brussels, Daimler, and INRIA). They concluded that a slower detector with multiple features plus motion (i.e., slower than one frame per second) performed well and detectors with a gradient histogram worked better for wide ranges of scales, while high occlusion levels made detection challenging. Angelova *et al*. [10] recently proposed a deep neural network-based (DNN) algorithm for pedestrian detection, which combines the ideas of a fast cascade and a deep network, and made C++/CUDA implementations available via [11]. However, owing to the high computational intensity, the above solutions cannot be integrated on off-the-shelf smartphones at present. Costea *et al*. [12] recently proposed a fast pedestrian detection method for mobile devices. The detection scheme relies on multi-resolution models applied over each half-octave, and employs multi-scale aggregation to obtain classification features. They reported an average frame rate of approximately 20 frames per second on mobile devices. However, this was limited to a rough contour resolution that enveloped the pedestrian within a square area. Moreover, its implementation is still undergoing improvements, and is not yet publicly available.

Obstacle detection constitutes a significant part in systems designed for pedestrian safety. Popular sensors for range-based obstacle detection systems include ultrasonic sensors, laser rangefinders, radar, stereo vision, optical flow, and depth from focus. However, none of these sensors can be viably employed so far. For example, ultrasonic sensors are cheap, but suffer from specular reflections and poor angular resolution. Laser rangefinders and radar provide better resolutions, but are more complex and expensive. In Smombie Guardian, the goal is to achieve pedestrian and obstacle detection on mobile devices (i.e., lightweight computation and inertial sensors), and measure accurate contours of obstacles with high fidelity to detect variations in image sizes based on user displacement. Thus, we adopt the monocular color vision approach recently proposed by Ulrich *et al*. [13]. This approach uses a single passive color camera, performs in real time, and provides binary images of obstacles at high resolution. Our implementation performed well in real time, provided high-resolution obstacle images, and operated in a variety of environments. Furthermore, the algorithm is very easy to train.

### User displacement tracking

To track user displacement, we can keep track of accelerometer readings. As a sub-task of dead reckoning (dead reckoning is composed of two sub-tasks, namely computing the user's displacement from accelerometer readings and continuously tracking changes in directions from compass and gyroscope readings), this is a well-studied area of indoor localization. Wang *et al*. [6] reported that double-integrated accelerometer readings provide unacceptable results for accurately obtaining a user's displacement. They further suggested that a user's physical displacement can be more accurately computed by multiplying step counts by the user's step size, which is a function of the user's weight and height. Noh *et al*. [14] further investigated user step size profiling, and reported that step sizes are not closely correlated with a user's height and weight, but rather with their step speed. In another area, Cho *et al*. [15] proposed AutoGait, which is a step size auto-calibration method on a mobile platform that trains a user's walking profile by effectively processing noisy GPS readings. Conveniently, Smombie Guardian does not require diverse step size profiles according to step speed (i.e., frequency), as a user exhibits a unique pattern when distracted by activities on a smartphone.

### Smartphone-aided situational awareness: Effectiveness

Situational awareness comprises the perception and comprehension of one's surroundings and the use this information for action. Owing to the high accessibility of smartphones and their ability to collect data from various sensors, a large body of research has introduced many examples of the effectiveness of using smartphones to enhance situational awareness in various contexts. For example, in the context of healthcare, smartphones have been used to provide situational feedback to stimulate self-management for people with diabetes [16], chronic and widespread pain [17], and people in smoking cessation programs [18]. In emergency scenarios, smartphones have been used to detect in-vehicle accidents through accelerometers, and to notify authorities (e.g., emergency responders) in a given geographical location [19, 20]. In line with these findings regarding the potential uses of smartphones to enhance situational awareness, we aim to investigate how Smombie Guardian can help to enhance the situational awareness of smartphone users and provide feedback as an alert.

### Users and system alerts

The benefits of notification systems include the rapid availability of important information, access to near-instantaneous communication, and a heightened awareness of the availability of personal contacts [21]. However, the design of notifications should proceed carefully, as it can undermine user experience. According to a survey by Appiterate, 68% of smartphone users decided to delete apps because they found their notifications annoying (https://bit.ly/2JiGWs0). Therefore, in the context of this study our goal was to provide users with situational awareness to recognize obstacles and prevent potential collisions, while making this as unobtrusive as possible.

## Application design

### Choosing alert mechanisms

Four types of alerts (e.g., border, pop-up, vibration, and sound) can easily be supported and triggered by commercial smartphones. As part of our design process, before implementing the app we conducted an online pre-design survey to examine the types of alert mechanisms that smartphone users would prefer (i.e., the most effective and unobtrusive). Note that our intention in this design probe was not to present a novel user interface for the feedback mechanism in Smombie Guardian. Rather, we intended to adopt the most preferable type of feedback in order to focus on the feasibility our algorithm and understand the user experience for the idea of Smombie Guardian.

The survey began with the following simple usage scenario:

Suppose that you are walking while reading an article on your smartphone. You focus too much on the smartphone screen, and become unaware of a person (or an obstacle) in your way. You may collide with the person (or the obstacle) if you keep walking and reading the article on your smartphone screen.

**Fig 1:**
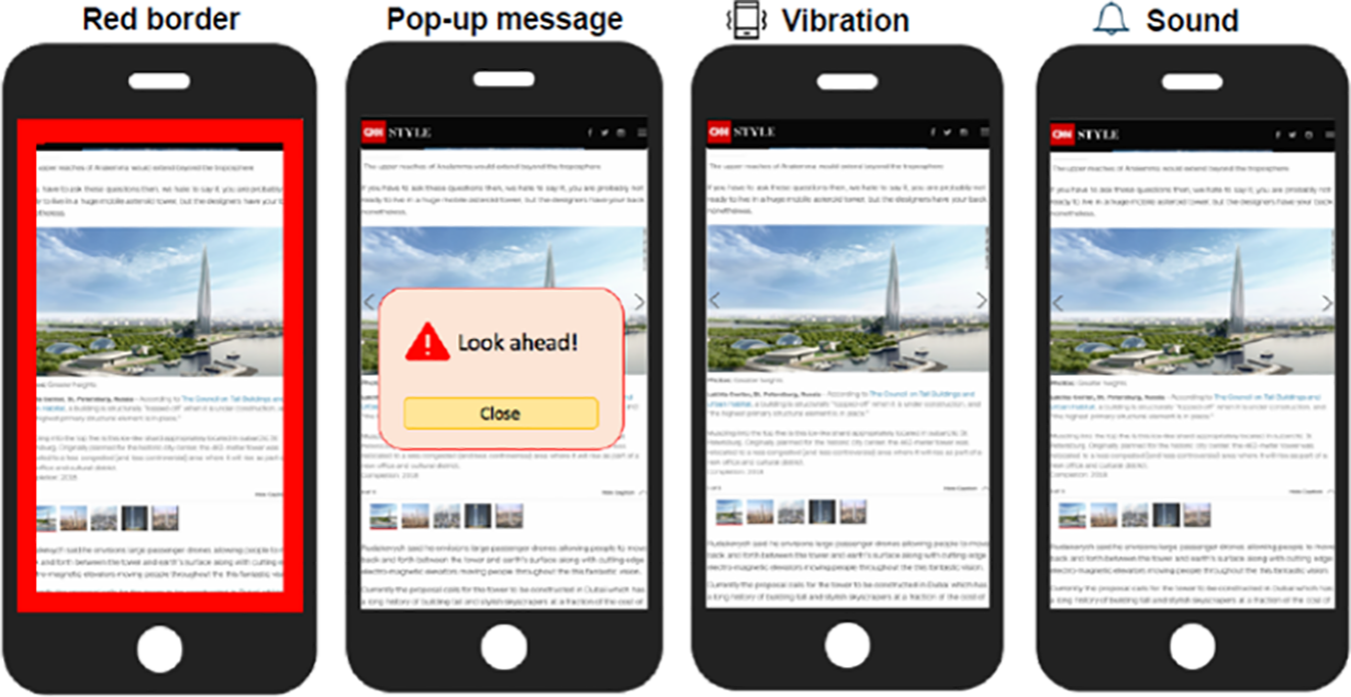
Four types of alert mechanism. Based on the results of our user study, we chose the two most preferred features (colored border and vibration) for the design of our app.

Next, the survey asked whether respondents had been in such a situation, and introduced the four alert types—a red-colored border (red border, interchangeably), a pop-up message, vibration, and sound (Fig 1). It asked the respondents to rate each alert type when generated by the smartphone based on three aspects (i.e., level of effectiveness, obtrusiveness, and preference).

We recruited university students through class announcements and mailing lists in March 2017. The survey took five minutes to complete, and we received 105 responses. Here, 75.2% of the respondents were in their 20s, and 24.8% were in their 30s. Because we ran the pre-survey at the university, 92.1% of the respondents were either undergraduate or graduate students, and 67.1% of the respondents were female.

The survey results provided us with a number of insights and design guidelines. First, a total of 67.3% respondents (50.0%—occasionally, 16.3%—sometimes, 1.0%—often) indicated that they had encountered a similar situation to the one described in the above scenario. Second, 32.7% of the respondents reported having experienced becoming unaware of their surroundings and of colliding with an object or a person. Third, regarding the alert type (summarized in Table 1), the pop-up method yielded the most beneficial result (4.13±0.98) in terms of effectiveness, and the second best result (3.24±1.25) in terms of preference. However, it also delivered the best result in terms of obtrusiveness (4.09±0.92), which makes it likely that users could end up turning off the feature. The border method showed the second-best results (3.69±1.08) in terms of effectiveness, poor results for obtrusiveness (2.84±1.12), and the best in terms of preference (3.68±1.12). Thus, it appeared to be a useful feature overall.

**Table 1:** Summary of 105 responses (Mean and SD) for four types of alert mechanism with respect to effectiveness, obtrusiveness, and preference (1: Strongly disagree; 5: Strongly agree).

Type	Effectiveness	Obtrusiveness	Preference
Border	3.69 (1.08)	2.84 (1.12)	3.68 (1.12)
Pop-up	4.13 (0.98)	4.09 (0.92)	3.24 (1.25)
Vibration	3.00 (1.20)	2.30 (1.04)	3.16 (1.20)
Sound	2.75 (1.20)	2.77 (1.14)	2.22 (1.01)

As our design rationale involved providing alerts (using at least two methods) to users in an effective and unobtrusive fashion, based on the survey results we decided to apply the border and vibration alerts in our app.

## Prototype design and implementation

### Prototype design

**Fig 2:**
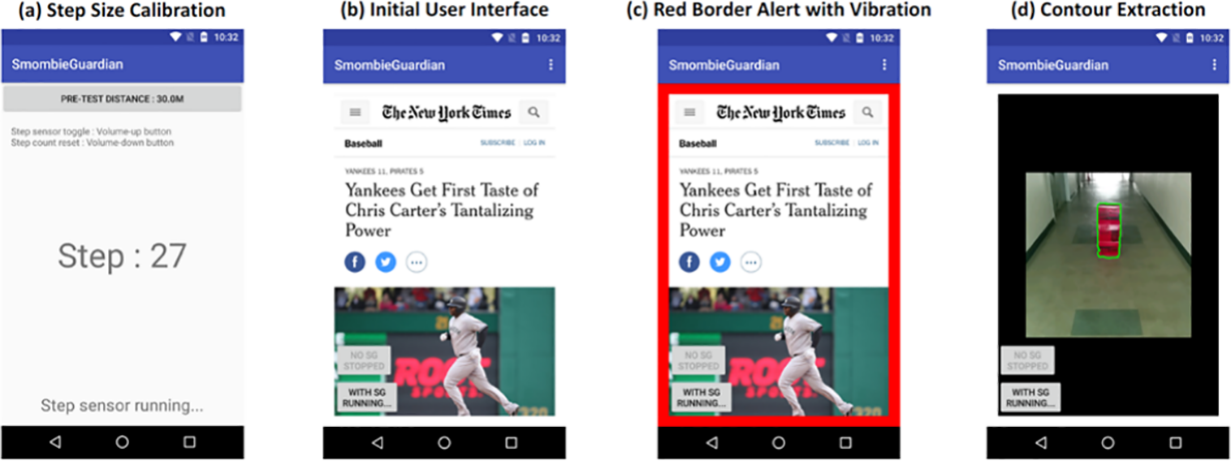
Screenshots of the Smombie Guardian (SG) prototype. In (a), each user can calibrate SG by creating a step size profile to improve the distance accuracy. When the user is about to collide with an obstacle, SG triggers a red border and a vibration (from (b) to (c)). SG extracts the contour of the obstacle based on color in (d).

Smombie Guardian is composed of two components. As shown in Fig 2(A), the first component comprises step size calibration. For evaluation purposes, we instantly obtained the step size of each user by counting the number of steps as a subject in our experiments walked along a fixed trail (i.e., 30 meters in our study setting). Having obtained the number of steps, we easily obtained the average step size of each user by dividing the fixed trail length by his/her step count. Fig 2(B) shows a screenshot of the initial user interface (UI), the second component of Smombie Guardian. Users manually activate and deactivate Smombie Guardian by using two toggle buttons (located at the bottom left of the screen). Note that we implemented Smombie Guardian as a foreground app for the purpose of the experiment. It could easily be implemented as a background service so that the user can freely use Smombie Guardian with other smartphone apps while walking. Fig 2(C) shows a screenshot of the red border alert triggered by Smombie Guardian when a user is about to collide with an obstacle.

### Implementation

It is fairly challenging to measure the distance between a user and an obstacle with a single-lens camera. To the best of our knowledge, no existing study has computed this distance with an acceptable accuracy. Instead of relying on a vision-based approach, we resolve to use the ratio of the user's displacement to the variation in image size to accurately estimate the size of an obstacle and the distance between it and the user. With this novel approach, we can compute the sizes of and distances to obstacles with a considerably high precision (average error with fixed displacement < 1%). Moreover, Smombie Guardian needs to notify the user of impending collisions in a timely manner, without consuming too much battery power. The following will explain the implementation details of our proposal to achieve these objectives.

#### Obstacle size and distance estimation

Recognizing the depth (or distance) of an object from a monocular image is a non-trivial and error-prone task [22]. A large body of research has been carried out in this area, but the only applicable approaches to smartphones are monocular cues and motion parallax [23]. The former uses cues of how humans recognize distances from monocular images (e.g., known object sizes, defocusing, and texture gradients) [24]. However, this approach requires machine learning techniques to process additional information in order to better estimate distances of target objects (i.e., deep convolutional neural fields [25], reinforcement learning [26], etc.), which are computationally challenging, and not applicable to off-the-shelf smartphones. On the other hand, the latter relies on optical flow (i.e., image velocity), and is widely employed as an approximation method for the motion fields in images. To infer the distance, differential techniques are employed, such as regional-based, energy-based, and phase-based schemes [27]. However, these cannot measure stationary obstacles, and require each pixel displacement (with a dense series of images) to be calculated (which is computationally intensive). Instead, our novel scheme accurately computes the distances to and sizes of both stationary and non-stationary objects with the help of the user's displacement.

**Fig 3:**
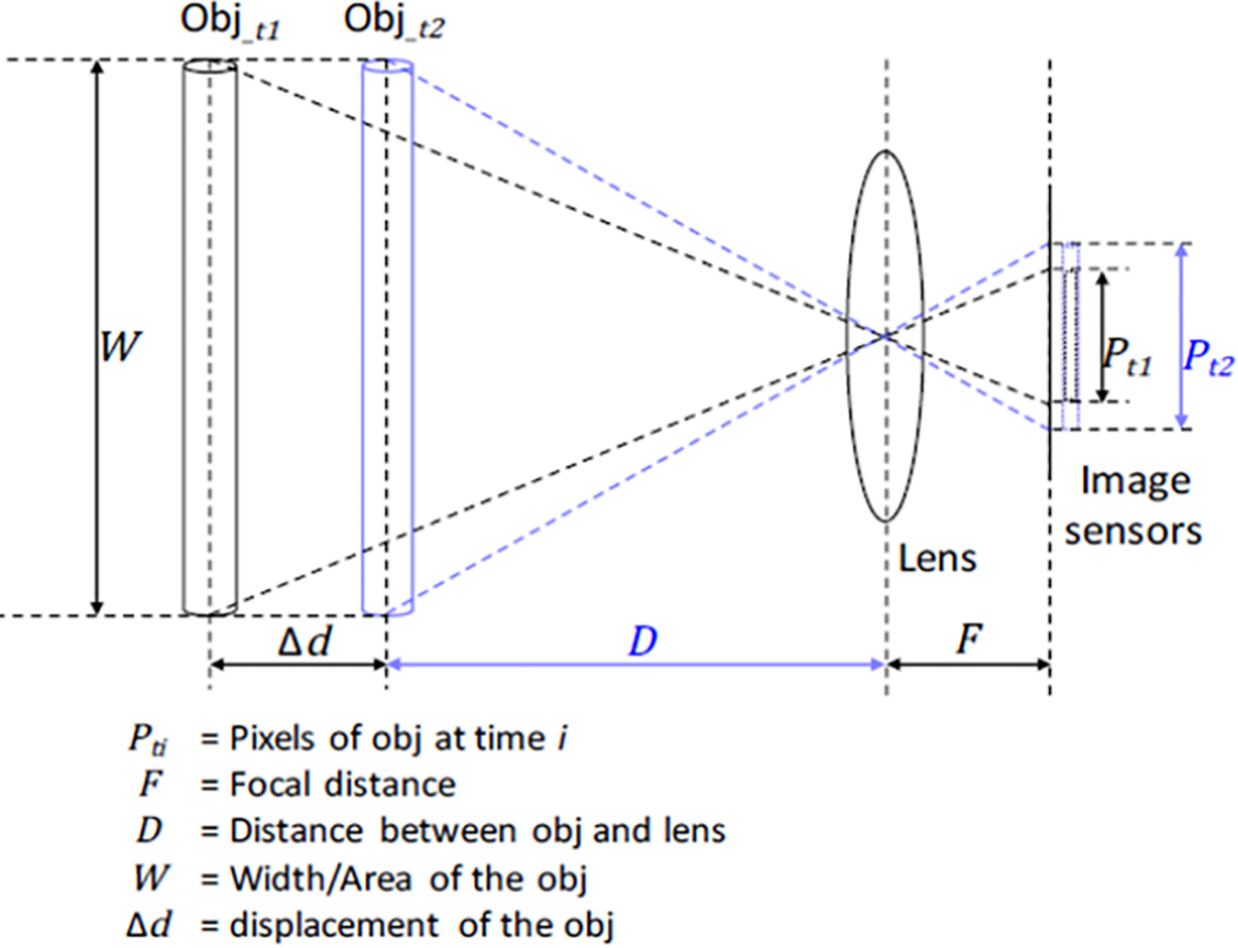
Triangle similarity of the single-lens reflex camera at *t*_*1*_ and *t*_*2*_.

**Fig 4:**
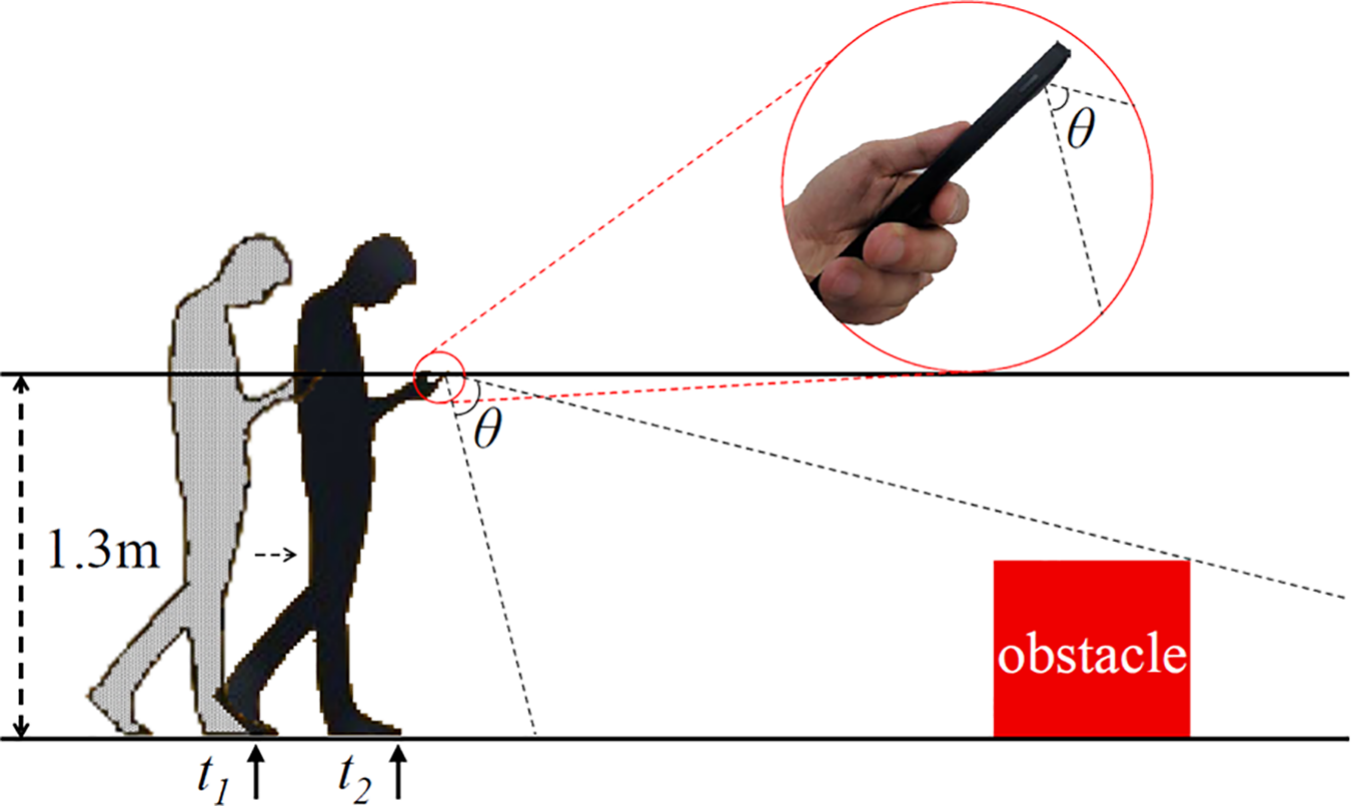
An obstacle that can be captured at each step (*t*_*1*_ and *t*_*2*_). The field of view (*θ*) was 69.4 degrees for the Nexus 5X (focal length of 26.6 millimeters) smartphone.

To obtain size of an obstacle and the distance between it and the user, we can use the triangle similarity of a single-lens reflex camera (Fig 3). For example, consider an object of unknown width *W* (or *area* in our implementation). We place it at some distance *D* from our camera. When we photograph the object using the single-lens camera, we can obtain the apparent width in pixels, *P*. As the focal length *F* of our camera is fixed (i.e., known), we have the equation *F/P = D/W*, which is used to calculate the distance between the camera and an object in an image. However, in this example there are two unknowns, namely *D* and *W*. As shown in Fig 4, to solve the unknowns in the equation we need at least two equations at different locations, namely *D* at time *t*_*1*_ and *D+ΔD* (user's displacement) at time *t*_*2*_, as the pixels *P* on the smartphone screen will be altered from *P* to *P+ΔP*. As we now have two equations for the two unknown variables, we can solve them with ease. Furthermore, to accurately compute the user's displacement (i.e., step size) and the variation in the image size on the screen (pixel variation before and after the step), two aspects must be considered.

First, we note that each user typically has a unique gait pattern (i.e., step size) as they walk while occupied by activities on a smartphone (e.g., texting, reading an article, or playing a smartphone game) [28]. Step profiling [14], which a GPS-based learning approach, requires a long training time to obtain an acceptable user step size. The Smombie Guardian can learn the user step size by tracking the outdoor displacement (only when the smartphone screen is on) through GPS with the help of a pedometer. Second, continuously tracking an object (i.e., contouring) and computing its area in a series of images (e.g., 15 frames per second) are both well-studied research problems [9, 12, 29]. However, carrying out these tasks on an off-the-shelf smartphone is challenging. To prove the concept of our design, we instead used a distinct color (as shown in Fig 2(D), with a red color in our field study) to obtain the contour and its area on a commercial smartphone screen, at a rate of least 10 frames per second. We implemented a prototype of Smombie Guardian on the Nexus 5X (released October 2015, where the app can be installed on phones with Android version 5.0 and above), and confirmed that we could obtain the contours of obstacles at 15 frames per second, which was sufficiently high to track variations in the contour sizes of obstacles in real time. It is also important to know the distance that can be covered by a rear-facing single-lens camera. As shown in Fig 4, when a user holds a smartphone (Nexus 5X in our setting) at a height of 1.3 meters, the distances covered by it are depicted according to the angle of the smartphone. Considering that the Nexus 5X is equipped with a 26.6-millimeter lens, its field of view is 69.4 degrees (i.e., θ in Fig 4). Thus, a smartphone angle of 44 degrees from the horizon is adequate to capture a 70-centimeter obstacle located at a distance of three meters from the camera.

#### Timely alert

In a study on drivers' reaction times, Johansson *et al*. [30] found that the corrected median of the resulting distribution was 0.9 seconds, and 25% of the considered group were estimated to have brake reaction times of over 1.2 seconds. Taoka [31] reviewed four studies concerning drivers' reaction times, and found that the median value ranged from 1.07 to 1.14 seconds, whereas the mean was between 1.14 and 1.30 seconds. Based on these results, we used the worst (or longest) time taken to generate alerts as a user approaches an obstacle (or vice versa), which was approximately 2 seconds. Considering that each step took 0.5 ~ 0.6 seconds as a user walks and the step size was between 0.6 ~ 0.8 meters, we set the default setting to 3 meters (approximately four steps). However, we also provide a knob for users to freely vary this distance based on their preferences.

#### Necessity-based activation

To minimize the smartphone's resource utilization and maximize the usefulness of Smombie Guardian, it can be easily implemented as a background service. In the background, Smombie Guardian is activated when a user is using their smartphone while walking. To be activated at the correct time, Smombie Guardian monitors the user's displacement via inertial sensors (i.e., accelerometers) and foreground apps that may require the user to attend to their screen. Smombie Guardian suspends its service if both conditions are not met. Through this conservative design, Smombie Guardian can save system resources (such as computation and battery power).

## User study design

The following research question has guided the design of our user study:

- How effectively does our app provide alerts to users to avoid potential collisions while using a smartphone and walking?

Our studies (both the prototype design and the main user study) were reviewed and approved by the Institutional Bioethics Committees of the Internal Institutional Review Board (IRB) at the Inha University.

### Design considerations

#### Content displayed on the phone

A study on the impacts of talking, texting, and listening to music on a phone [32] indicated that texting, which involves communication interchange as well as reading and typing, is more cognitively distracting and demanding than talking, and poses a higher risk of injury. Similarly, Lamberg and Muratori [33] studied changes in the gait velocity and trajectory of walkers when they interacted with a mobile device. The result showed that participants who texted while walking moved 33% slower and deviated from their intended course 61% more often than those who did not. Under the same circumstance, we asked all participants in our experiment to read the same news article. As with texting, we believe that reading a news article will make users sufficiently distracted to not focus on their surroundings.

#### Pre-test

Walking patterns and speed tend to vary from person to person. Because it would be challenging to consider different walking patterns and speeds in designing our app, we ran a small study to measure a common range for these parameters before conducting the main user study. We placed five obstacles along the same path that we used for the main study. We invited seven participants (three undergraduate and four graduate students, average age 28.1, two females and five males). Following the demo and pre-test, the participants mentioned that the app had functioned correctly, generating alerts when they had felt obstacles close to them. On the whole, the design choices of Smombie Guardian and the pre-test results allowed us to focus on studying the user experience and perception of the app, as well as suggestions and implications for the design of an alert-based mobile system.

#### Study procedure

The main study was conducted in May 2017. Before conducting the experiment, participants were first asked to read the informed consent document, which explains the goal and procedure of our survey study. Only the participants who read and agreed with our study procedures and requirements and provided written consent for the user study and publication could begin answering the survey.

**Fig 5:**
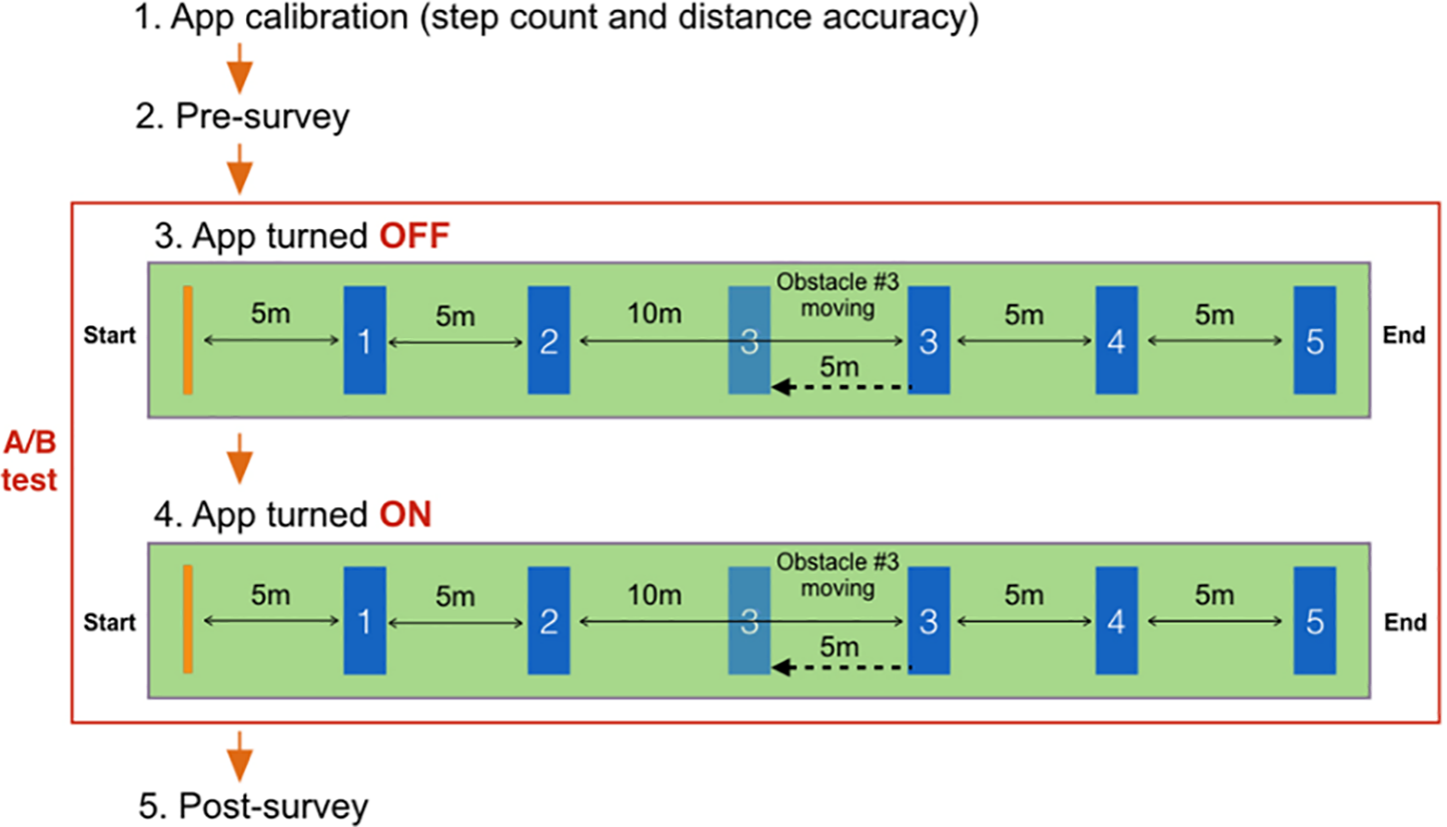
Procedure of the main user study. We designed a within-subject user study. We first (1) calibrated Smombie Guardian to improve the step count and distance accuracy for each participant. After (2) completing the pre-survey, the participants were asked to read a news article on a smartphone screen and walk along a path (3) without the proposed app turned on. Then, they were asked to walk the same path (4) with it turned on. Five obstacles were placed along the path. Obstacle #3 was moving toward the participants. Finally, the participants (5) completed the post-survey.

We designed a within-subject study, in which every participant first walked to the five obstacles sequentially without turning on Smombie Guardian, and then walked to the same five obstacles with Smombie Guardian turned on. As shown in Fig 5, the study consisted of five steps.

First, to improve step count and distance accuracy for each participant, we calibrated Smombie Guardian by asking each participant to walk 30 meters (normally generating 35 ~ 45 steps). Second, we asked the participants to complete the pre-survey, containing questions concerning their demographics, the duration and frequency of their smartphone usage, and their experiences of colliding with objects or people while preoccupied with their smartphones. Third, we asked the participants to read a news article on their smartphones while walking along a path, and dodge obstacles when they noticed them. Smombie Guardian was turned off during this step. Fourth, we asked the participants to turn on Smombie Guardian and repeat the above step. Before beginning the walk with Smombie Guardian, we asked the participants to take 10-minute break in order to minimize the learning effect. Once they had completed this, we asked them to summarize the contents of the article that they had read, to ensure that they had focused on reading it while walking. Finally, we asked the participants to answer the post-survey, containing questions about their experiences (e.g., effectiveness, usefulness, and obtrusiveness), and soliciting feedback concerning the design of Smombie Guardian (and more broadly alert systems for smombies). We validated the questionnaire in the survey prior to testing it on the participants. The study lasted for approximately 15 minutes, and we offered a small compensation (a $5 gift card) to the participants for their time.

## Results

### Participants

We completed the user study, which involved 74 participants, over 10 days. Once the participants had finished walking along the 30-meter track, we asked them to describe the content they had read on their smartphone screens. All participants correctly answered and summarized the article, which showed that they had focused on reading it while walking along the path. Most participants in the study were in their 20s (71; 97.3%), and three participants were in their 30s.

Forty-nine participants (67.1%) were male. As expected, all were familiar with the use of smartphones. Over 85% (63 participants) had been frequently using them for over four years (constant use for 31 participants, frequent use for 32 participants). Regarding the frequency of smartphone use while walking, eight participants (11.0%) mentioned that they normally did this, 32 (43.8%) participants said that they had often done so, and 24 (32.9%) claimed that they had sometimes used their smartphones while walking. This implies that many of the participants may act like smombies in many situations. Regarding their experiences of collisions, 30 participants (40.5%) said that they had previously come very close to colliding with a person or object because they had been preoccupied with their phone.

### Collision analysis

#### All participants

**Fig 6:**
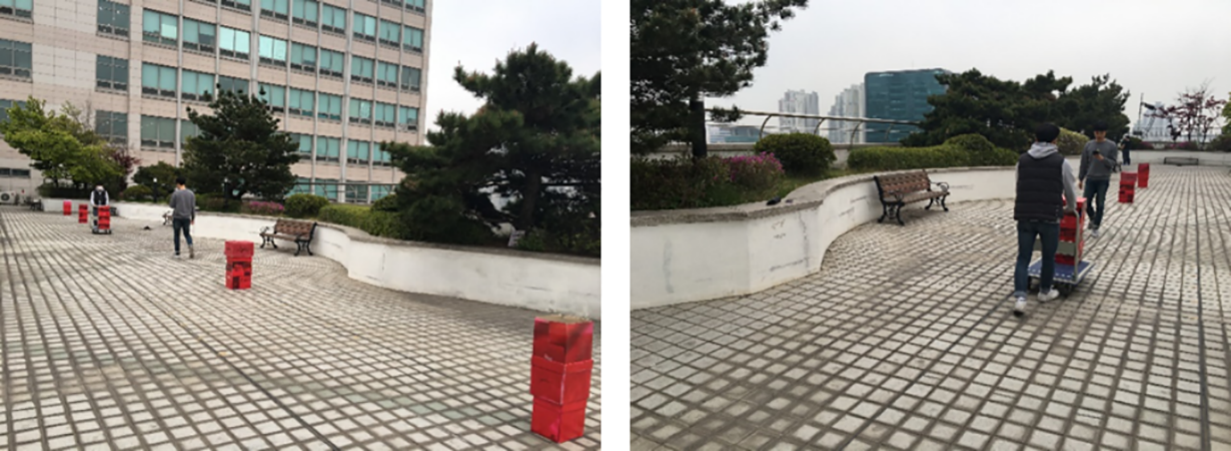
Snapshots of the Smombie Guardian field test: (Left) Overall view of the 30-meter track and the locations of obstacles. (Right) A participant using the Smombie Guardian encounters a moving obstacle approaching (Note that the individual in this manuscript has given written informed consent (as outlined in PLOS consent form) to publish these case details).

To assess the usefulness of Smombie Guardian, we conducted a large-scale field test (involving 74 participants) on our campus. Fig 6(A) shows the field test settings. We placed five obstacles (four fixed and one moving toward the participants) on the track. Fig 6(B) shows a participant confronting an obstacle moving toward them.

**Fig 7:**
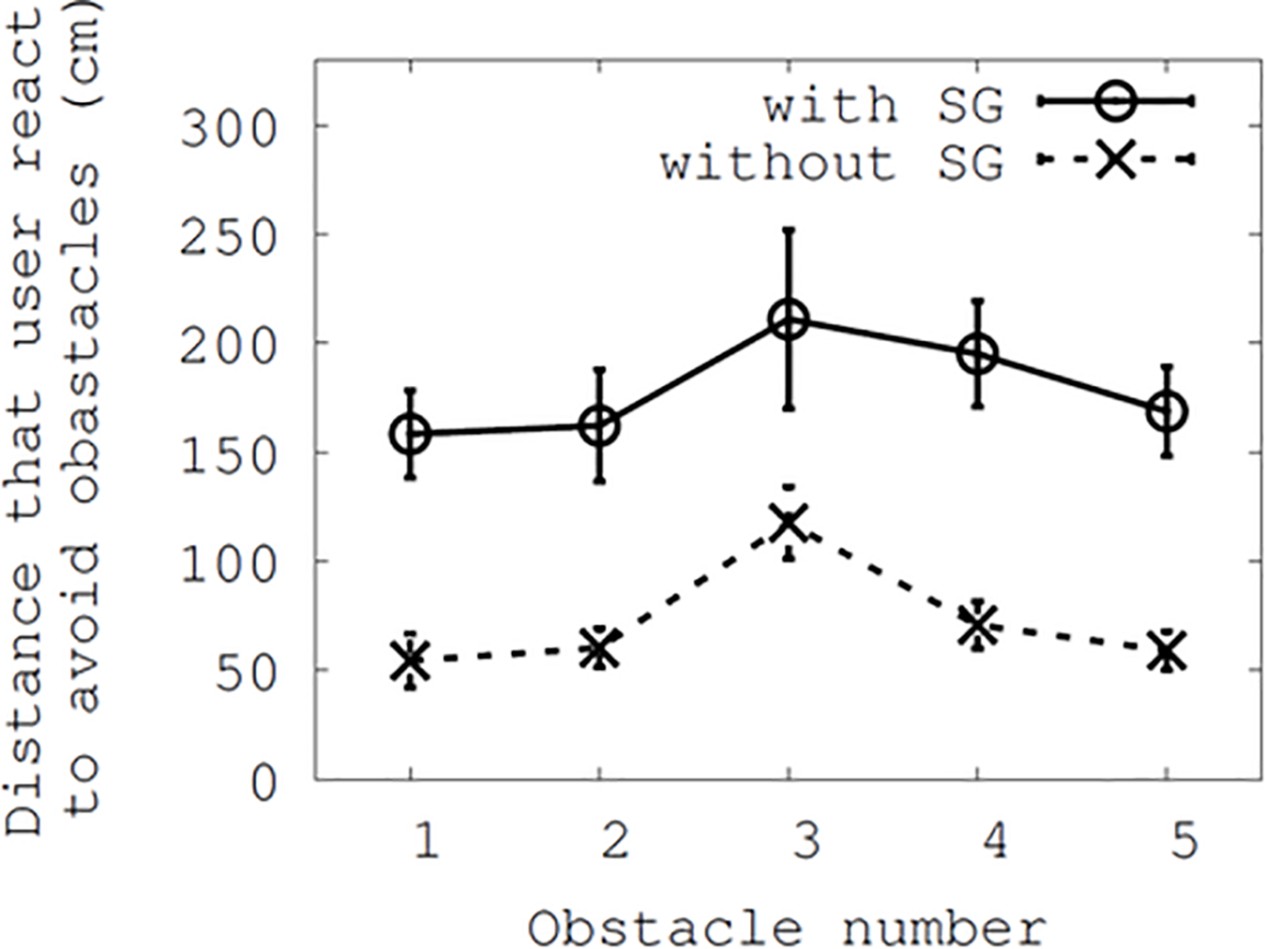
Collision avoidance distance: *With* and *without* Smombie Guardian, as a function of the obstacle.

Fig 7 shows the measured distances at which the participants reacted to the five obstacles. The two conditions of having Smombie Guardian on or off are plotted with a 5% confidence interval. As shown in the Fig 7, users with Smombie Guardian acted to avoid obstacles approximately one meter earlier than those not using Smombie Guardian. Statistically, the distance without Smombie Guardian is significantly different from that with Smombie Guardian for all obstacle numbers at p < 0.001 from the ANOVA analysis (F(1,124) = 12.41). According to studies on drivers' reaction times [30, 31], drivers may require reactions times ranging from 1.14 to 1.30 seconds to safely avoid possible collisions. Considering that each step takes between 0.5 and 0.6 seconds and their sizes range from 0.6 to 0.8 meters, users may be required to become aware of obstacles at a distance of 1 to 1.6 meters. This shows the usefulness and effectiveness of Smombie Guardian with respect to helping the participants take actions in advance. It was also interesting that each error bar for Smombie Guardian was wider than the corresponding one without Smombie Guardian. That is, without Smombie Guardian reaction distances were convergent, whereas with Smombie Guardian these distances varied by participant. However, the overall distance when using Smombie Guardian was always longer (106.22 cm on average) than that without it.

It is worth noting that the participants both with and without Smombie Guardian exhibited similar behaviors in response to obstacles. It appears that participants avoided the first obstacle both with and without alerts. Then, the participants unconsciously anticipated the second obstacle earlier, as they saw it while avoiding the first one. Regarding the third moving obstacle, the participants appeared to be more cautious, and hence avoided it sooner than the other obstacles. For the fourth, it appeared that they were still influenced by this caution. However, they avoided the last obstacle later than the previous one (#4). They may be because they felt safer after having repeatedly avoided prior fixed obstacles.

**Fig 8:**
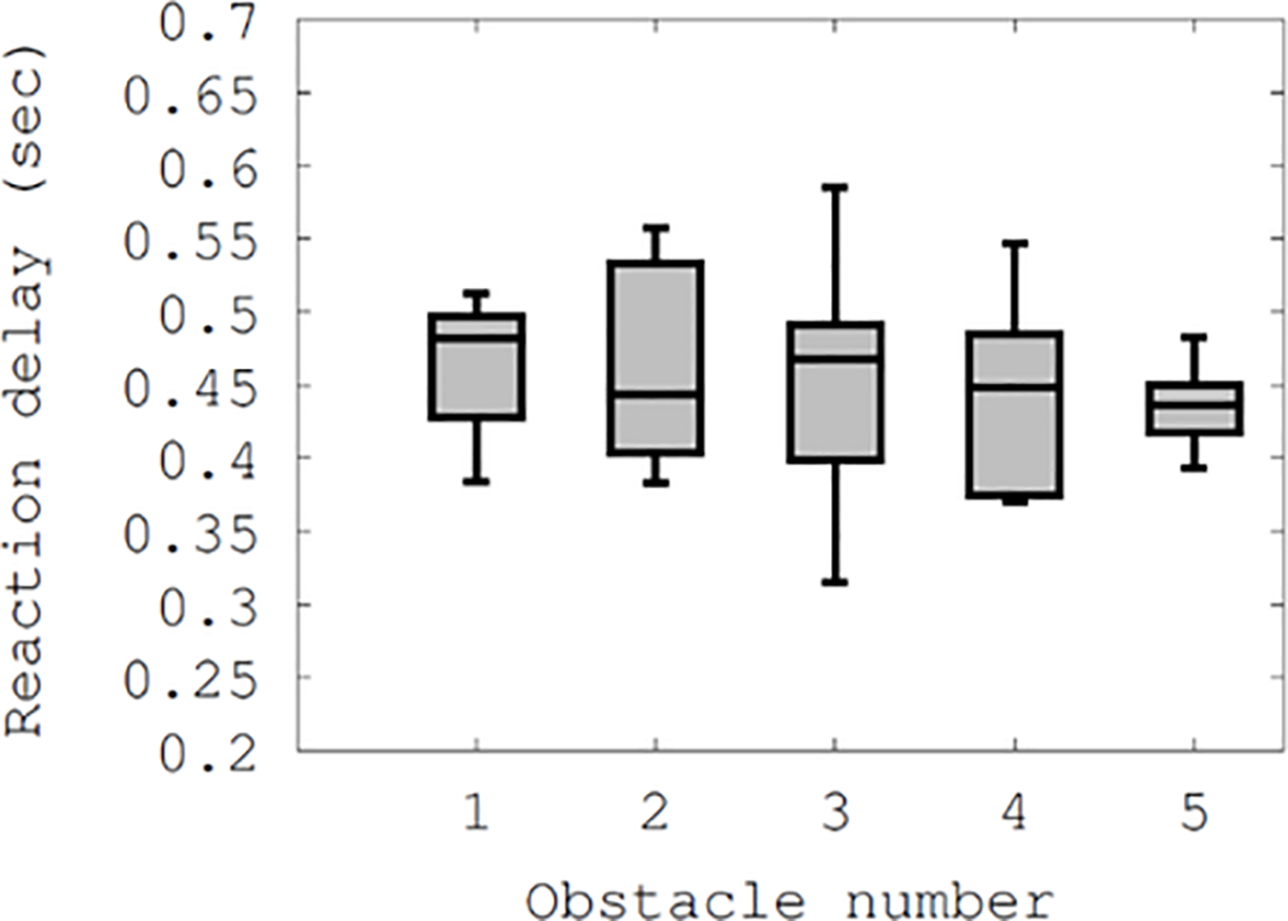
Users' reaction delays as a function of the obstacle.

As shown in Fig 8, we further analyzed the wider error bars with the use of Smombie Guardian in Fig 7, and found that participants had different reaction time gaps (ranging from 0.31 seconds to 0.58 seconds for the moving obstacle #3), where time ticks were measured between timestamps of warning notifications issued and actions performed (i.e., distinct accelerometer changes along the x-, y-, and z-axes at the same time). In Fig 7, the granularity of the time unit was 1 millisecond. Hence, we obtained a microscopic view for each obstacle. It is worth noting that the maximum of 0.58 seconds might not be the sole cause of the error bar of approximately 1 meter in Fig 7, considering that participants step sizes were between 0.6 and 0.8 meters. One reason for this was the granularity of alerts. Smombie Guardian checked alert-triggering conditions whether or not a participant entered the threshold distance (3 meters in our default setting) relative to an obstacle. Thus, one participant could trigger the condition very close to the threshold while another would trigger it far from this. We also noticed that some participants ignored the notifications, as they may have believed that they had occurred earlier than necessary to avoid the obstacles. This might have contributed to the wider error bar with Smombie Guardian than without it in Fig 7.

#### User groups (collision experience)

**Fig 9:**
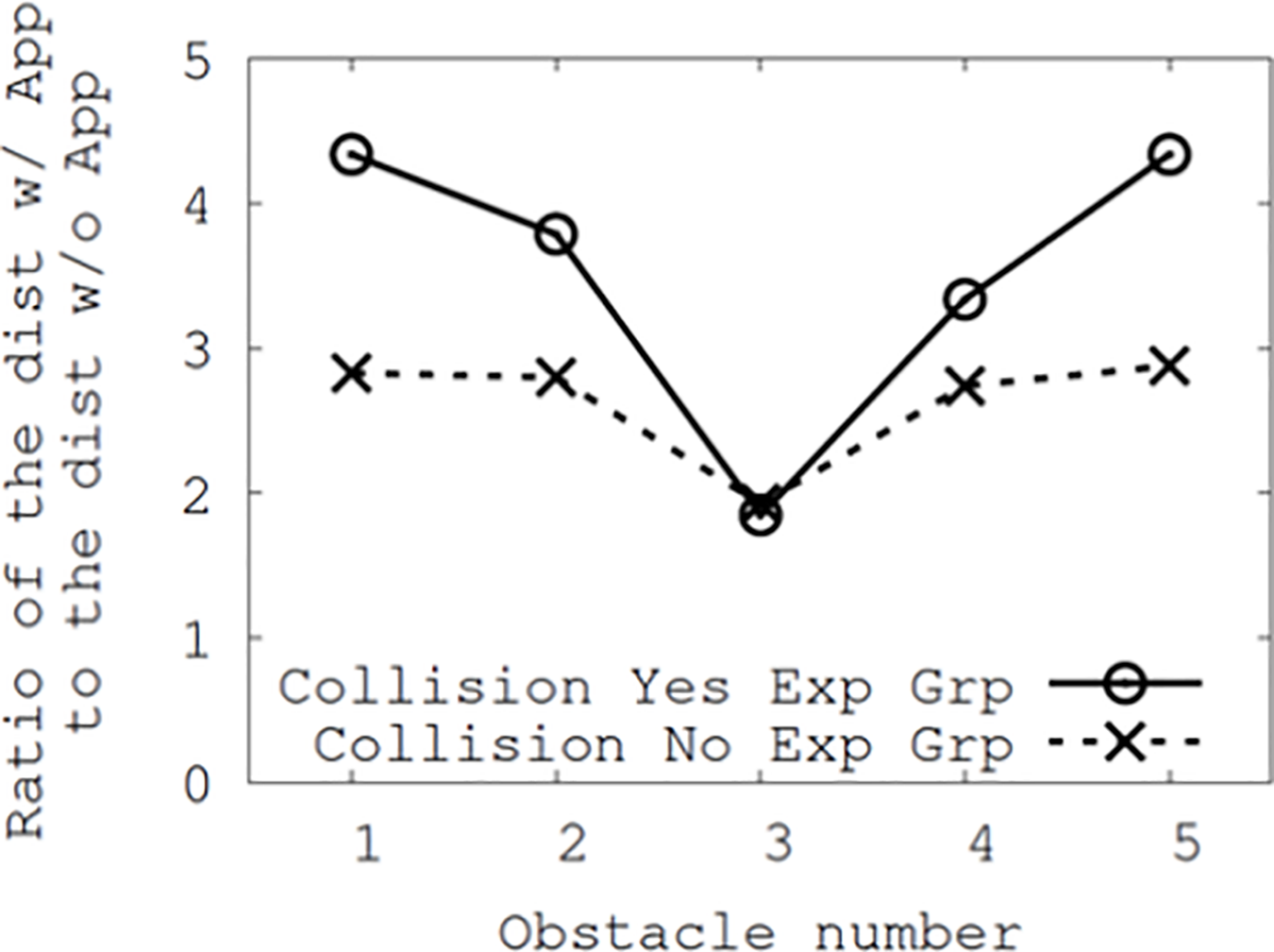
Ratio of the distance when the app was turned on compared to that when it was turned off.

We further analyzed the differences in collision distance by user group. In the pre-survey, we asked the participants whether they had experienced (or almost been involved in) collisions while preoccupied by their smartphones. Based on their answers, we clustered them into two groups (40.5% were in the collision group). We then calculated the distances between each obstacle and participant when they reacted. Fig 9 shows the ratio of the distance when the app was turned on compared to that when it was turned off. A higher positive ratio implies a greater reduction in the distance when the app was turned on. In Fig 7, it is shown that the participants tended to react more quickly when the app was turned on. When we considered this with respect to group differences, the group with prior experience of collisions recorded a higher ratio. This indicates that while Smombie Guardian significantly increased the distance at which a reaction occurred in general, it exhibited a greater influence on such changes in reaction times for the group with collision experience. In other words, Smombie Guardian helped participants who had prior collision experience to react to obstacles both earlier and more quickly (because the ratio of the distance with the app turned on to that without the app was greater for the collision group than for the non-collision group), which could potentially prevent them from experiencing a collision. In other words, Smombie Guardian appeared to work more effectively for people who had previous collision experience. For the inferential statistics, we observed a marginally significant difference at obstacles #1 and #5 (*F*(1,124) = 1.75, *p* < 0.10). However, when we compared the difference between the two groups without considering the obstacle numbers, we observed a significant difference (*F*(1,124) = 4.12, *p* < 0.02).

**Fig 10:**
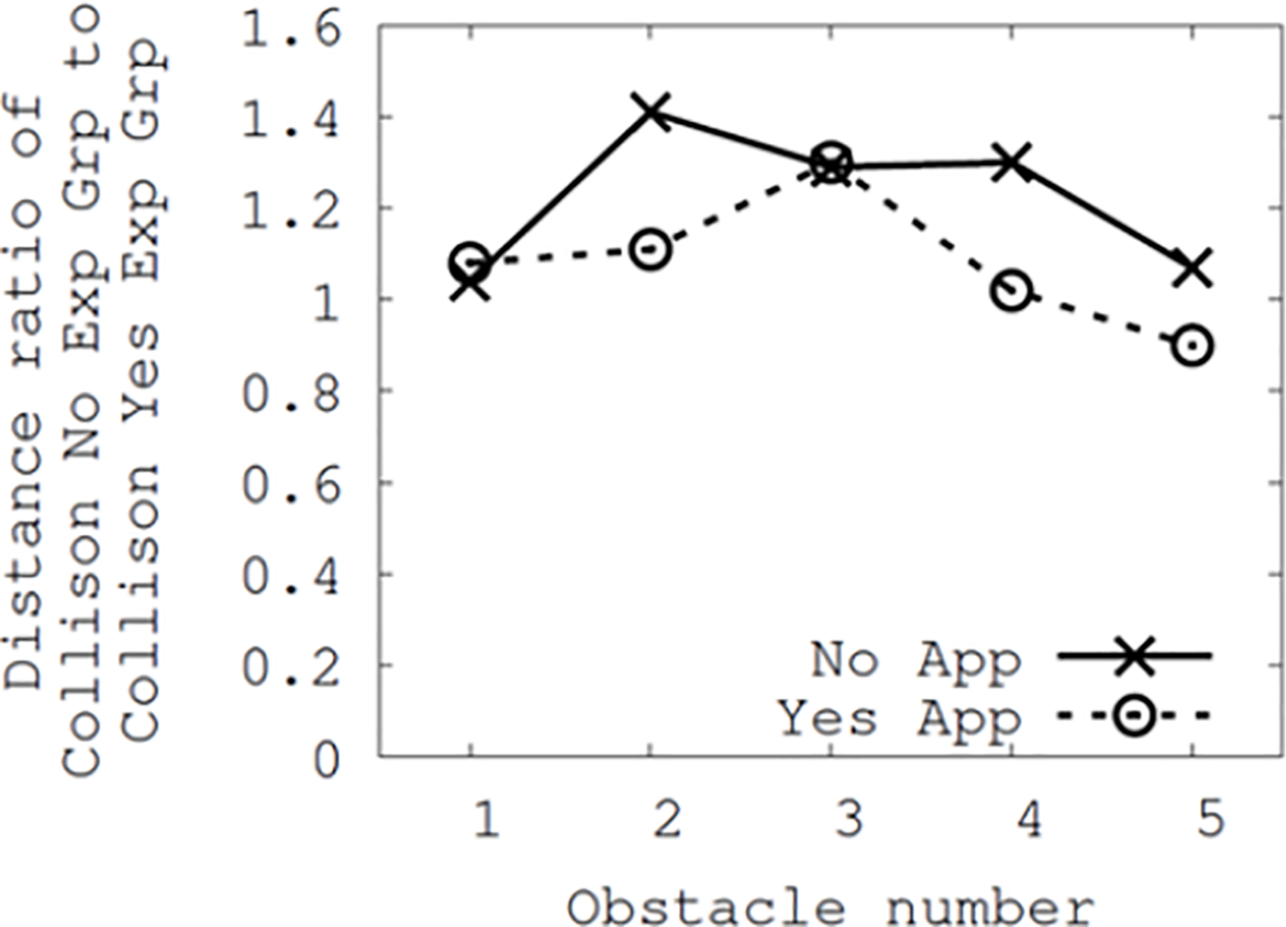
Ratio of the distance from the participants who did not experience collision (no exp group) to the distance from the participants who did (yes exp group).

Fig 10 shows the ratio of the distance for the participants who had not experienced prior collisions (namely non-the collision group) to that of the participants who had (the collision group). Because the y-axis represents the distance ratio, a value closer to 1.0 indicates that the distance between the two groups is smaller. Therefore, the difference in the distances between the two groups was smaller when Smombie Guardian was used than when it was not used. This further indicates that Smombie Guardian could be effective for people who have previous collision experience (or people who may be less aware of their surroundings when using their smartphone or focus too much on their smartphone while walking). Because the final results of Fig 10 were based on the average ratio between the collision and non-collision groups, there were no inferential statistics.

Overall, both Figs 9 and 10 demonstrate the results for the two user groups regarding the use of Smombie Guardian, as well as the effectiveness of the app on the participants with prior collision experience.

In addition, note that we can observe considerable corrections in both Figs 9 and 10 in cases involving fixed obstacles, #1, #2, #4, and #5, but this is not the case for the moving obstacle #3. We again recruited participants from both groups to understand the differences, and found that they easily noticed the moving obstacle beforehand, as it had been approaching with a recognizable sound (the sound of a cart being drawn). This led to identical obstacle avoidance behavior in both groups.

### User experience

We measured the efficiency, usefulness, and obtrusiveness of the app in order to understand the user experience. The following questions were used to understand each case.

- *Efficiency*: To what extent do you find the alert efficient (1: Not efficient at all, 5: Very efficient)?
- *Usefulness*: To what extent do you find the alert useful (1: Not useful at all, 5: Very useful)?
- *Border*: I found the border notification obtrusive while using the app (1: Strongly disagree, 5: Strongly agree).
- *Vibration*: I found the vibration notification obtrusive while using the app (1: Strongly disagree, 5: Strongly agree).

**Fig 11:**
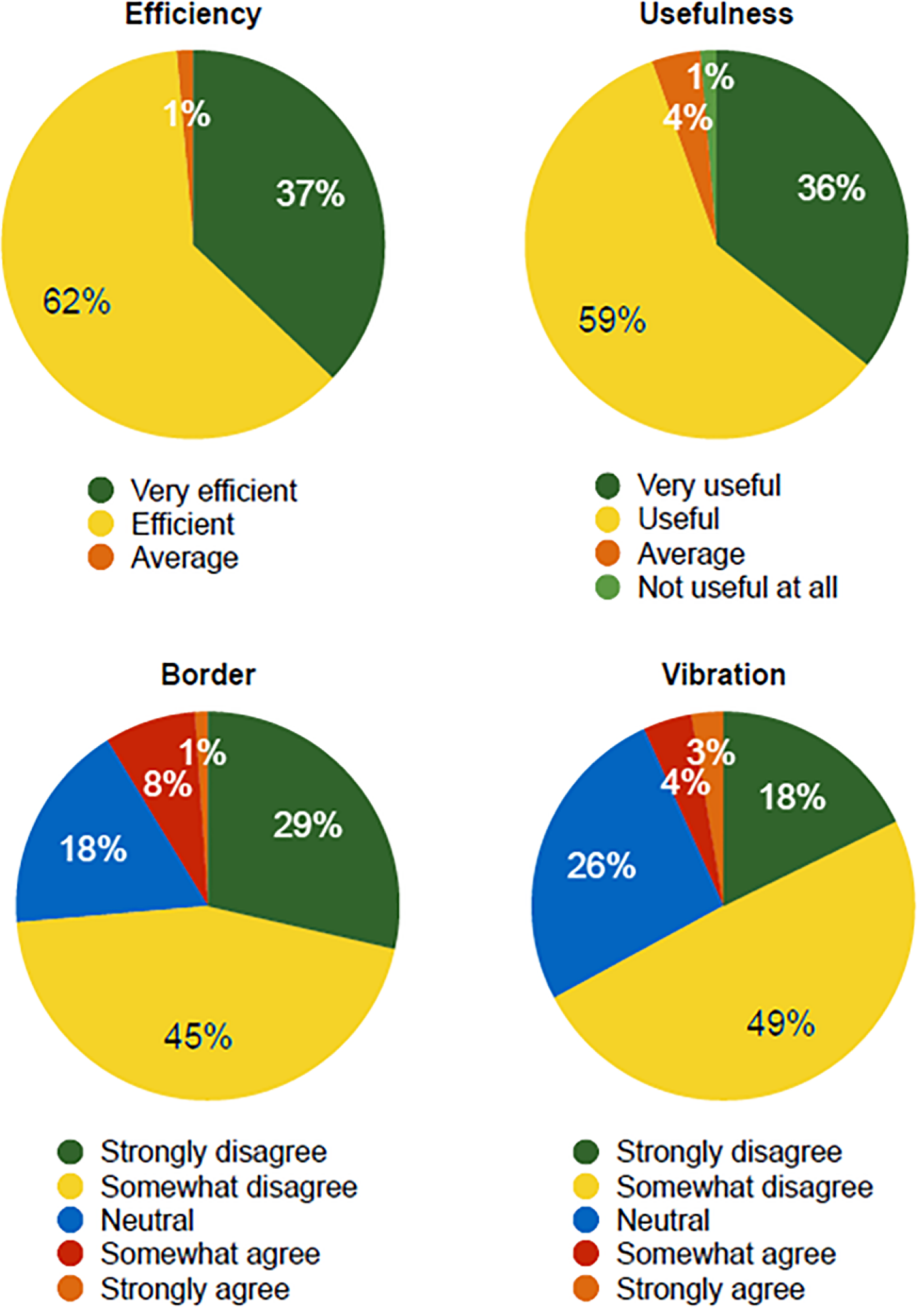
Efficiency (top-left) and usefulness (top-right) of the app; obtrusiveness of the app (bottom-left: border; bottom-right: vibration). Note that for obtrusiveness, a greater disagreement implies less obtrusiveness.

As shown in Fig 11 (second left), the participants' evaluations of the efficiency and usefulness of the app were high, with 37% rating it at 5 (very efficient) on efficiency and 36% at 5 (very useful) on usefulness. The average efficiency and usefulness values were 4.35 and 4.27, respectively. There were two types of obtrusiveness, namely the border and vibration alerts. As shown in Fig 11 (second right), for the border 74% of the participants thought this was unobtrusive (the sum of strongly disagreeing and disagreeing responses), and 67% thought that the vibrations were unobtrusive. While it appears that two-thirds of the participants did not find the alert feedback to be obtrusive, some had had the opposite experience (9% for the border and 7% for the vibration). Overall, the participants' ratings regarding the effectiveness and usefulness of Smombie Guardian were high, and they were low concerning its obtrusiveness. We received many positive responses. Most participants opined that smartphones should provide systematic support of the kind offered by Smombie Guardian, as they encountered many smombies in everyday life, and sometimes had unintentionally tended to act in a similar manner.

### Experience and design feedback

In the post-survey, we asked two open-ended questions. The first asked about the participants' general experiences of the proposed app, and for their feedback relating to it. In line with the high ratings regarding the efficiency and usefulness of Smombie Guardian, almost half of the participants (38) opined that the app provides a useful feature for smartphone users. Of these, 11 mentioned that they liked the app because it generated alert messages early enough for them to act. *I was able to easily notice obstacles*, *as I received the messages two-three steps ahead compared to the case where I had not used the app* (P27). *I was impressed that the app captured objects far from me and generated alarms* (P34). Four participants said that they had been in similar situations, and found the app to be very useful. *I use my smartphone while walking*, *and it seems useful to avoid obstacles* (P15). *I have been seeing a growing number of people*, *including myself*, *preoccupied with their smartphones while walking; in this sense*, *the app seems useful*. (P43).

At the same time, 13 participants shared some problems they had encountered while using the app. Eight felt that the app provided alerts much earlier than they had expected. This relates to a design implication: the app should allow users to customize the type and frequency of alerts. Two participants mentioned that having only one notification type (either border or vibration but not both) would suffice.

Regarding the design feedback for the app, Table 2 summarizes the five main categories (including others) of user responses. First, a total of 18 participants expressed a desire to be able to customize the alert features. For example, choosing an alert type (either border or vibration); setting the color, brightness, or duration of the border alert; and setting the strength of the vibration. One participant who seemed to prefer vibrations wanted the strength of the vibration to vary automatically depending on the distance between the user and an obstacle. *It might be useful to have a stronger vibration for incoming objects* (P53). Second, 13 participants mentioned a desire for the system to be able to identify more objects, so that it might reflect more realistic scenarios. The current version of Smombie Guardian can capture multiple objects, but when generating alerts the location of an object should be aligned with the direction in which the user is walking. As one participant said, *I am a bit worried about receiving so many alarms if I use this feature while I am in a busy*, *crowded area* (P46). Multiple design considerations are involved in handling multiple objects to generate alerts, and we discuss these below. Third, related to the second category, four participants mentioned that they found our study environment to be simple. They wanted the app to be tested in more complex (more realistic) environments, with more people standing, walking in the same direction, and passing by. *I wonder how the app would work if it captures multiple objects or people at the same time*. *Will it constantly generate alarms*? (P45). Fourth, two participants mentioned user interface-related aspects of the app. For example, one participant wanted to see the distance between the object and the user, and another participant provided a general comment on the interface asking for the option to change the width of the border.

**Table 2:** Summary of design feedback.

Category	Count
Customization	19
Camera angle	13
Various environments	4
User interface	2
Others	2

## Discussion

In this paper, we proposed the design of Smombie Guardian, which leverages a user's walking pattern and computer vision technology (allowing us to run the app directly on commercial smartphones without adding extra hardware). We described how it helps smombies immersed in smartphone-related activities (e.g., texting, web browsing, watching videos, and gaming) to safely avoid impending collisions with other pedestrians or obstacles (e.g., lamp posts, benches, and signboards), by notifying them using timely and unobtrusive alerts. In the user field study, we received highly positive feedback from the participants, many of who want the app to be embedded on off-the-shelf smartphones in the near future.

### Study implications

The following are the insights gained from our experience of developing Smombie Guardian and the user field study.

#### Effectiveness versus obtrusiveness

To inform users of impending danger, we used four types of alerts (border, pop-up, vibration, and sound) that can be easily supported and triggered by commercial smartphones. Through our pre-design survey, we determined the effectiveness and obstructiveness of each alert type. We also noticed that higher obtrusiveness increases user irritation, and can lead to users disabling the app. We found that people generally felt that the border and vibration alert types were effective and unobtrusive. However, users’ preferences for receiving alerts appeared to be quite diverse in terms of the color and size of the border, and the strength and duration of the vibration. Some users may prefer more obtrusive alerts (e.g., pop-up messages), because these can be more effective for interruption. Thus, offering user customization of alert types and the details of each alert type would be necessary for the success and long-term adoption of this service.

#### Technological capabilities versus user expectations

Accuracy is the most crucial factor affecting the success of the system. In the context of our work here, accurately estimating obstacle sizes and distances from the user are important system performance parameters, because the misdetection of obstacle sizes and distances leads to false positives (i.e., false alerts and early alerts) or false negatives (i.e., warning failures). To this end, we continue tracking the ratio of the user's displacement compared to the image size variation in order to accurately estimate obstacle sizes and distances from the user. This allows us to accurately and swiftly compute obstacle sizes and distances in real time.

An early alert may give the user an impression of a false-positive warning, while a later alert might not be very helpful. However, according to the results of the study, this also varies by person. Some users may want to receive alerts early, while others may want to receive them when the object is detected in the critical zone. To satisfy these varied demands, systems can be designed to allow users to choose the preferred distance to objects at which alarms are triggered, users' walking patterns (e.g., step size and distance), times, geo-locations (e.g., obtaining a level of crowdedness), or demographics (e.g., gender and age) can be collected and stored in a central server (i.e., cloud). While these types of data are continuously collected and stored, we can use state-of-the-art machine learning and deep learning techniques to train the collected data, build robust models, and generate the preferred settings (e.g., distance, alert frequency, and strength) for each smartphone user and improve the models as more data are collected.

Another important consideration, which was also expressed by many participants, is the app's ability to capture multiple objects of different sizes, approaching from multiple directions, and notify users accordingly. The current version of Smombie Guardian can capture multiple objects, but they must be within the field-of-view of a single-lens camera, which determines the perspective and the area covered by the lens. As smartphone technology is evolving over time, its ability to capture objects at a wider angle will become possible. As an example, recent smartphones (e.g., Apple iPhone 7 Plus, LG G9, and Huawei P9) are equipped with dual rear-facing cameras: one with a telephoto lens and the other with a wide-angle lens. Using both lenses, smartphones can capture more content in a single photo. With improved technological capabilities, it is important to set criteria (thresholds) to determine whether objects approaching from different directions (i.e., from the left or right) are likely to collide with users, as well as when alerts should be triggered.

#### System resource management

While policing user safety, continuous image processing at 15 to 20 frames per second expends a considerable amount of energy of a smartphone, which is problematic (and could lead to the user turning off the service). To this end, the app needs to be able to carefully consider a user's situation and be activated as needed. In the case of Smombie Guardian, users can turn it on or off. However, system-level automated management would also be desirable. Depending on the time and location of smartphone use and the user's walking pattern, the system could be designed to automatically switch on and off.

### Limitations and future work

Although our study provided a number of important insights, it has a few limitations that will be addressed in our future work.

First, the study conditions were fairly simple, as the sizes and shapes of the obstacles were constant, and only five obstacles, placed in a row, were considered. This may not reflect more complex scenarios, where many people are using their smartphones at busy locations, such as downtown and subway stations. Our next step will be to improve Smombie Guardian to more accurately and reliably recognize multiple objects of different sizes and shapes, and determine whether objects are approaching a user's critical zone. We plan to further study the efficiency of Smombie Guardian in a more complex environment, by considering a mix of multiple static and moving objects of various sizes and shapes. Once we are satisfied with the performance of the app, we will ask users to use it in a real environment.

Second, while we believe that humans and obstacles are important objects that should be detected by Smombie Guardian, perhaps more critical are bikes and automobiles, which can more likely lead to serious or fatal accidents. As these objects move considerably more quickly than humans, Smombie Guardian should be designed to quickly recognize these and warn smartphone users to avoid collisions. Adib et al. proposed WiTrack [34], which leverages a technique called frequency modulated carrier wave (FMCW). This technique maps differences in time to shifts in the carrier frequency (i.e., frequency sweeping), because such frequency shifts are easy to measure in radio systems by looking at the spectrum of the received signal. It has been reported that this technique can localize the center of a human body within a median of 10 to 13 cm in the x and y dimensions and 21 cm in the z dimension. Smombie Guardian could adopt this technique to localize approaching objects in all directions. However, this presents three challenges: 1) accurate and fast signal processing of the received radio wave in the smartphone kernel; 2) a limited spectrum of the WiFi chipset on a smartphone compared to the USRP frontend; and 3) the user’s mobility/displacement cancellation. These challenges will be addressed in our future work. In addition, the alert mechanism used in our user study may not be generalized. As we mentioned previously, allowing users to select the best alert type for them would be more desirable.

Third, Google Project Tango, released in 2016, can compute an object’s size and the distance from an obstacle. However, this technology requires both depth- and motion-tracking sensors, which are not commonly available in most commodity smartphones (they are only available in the Phab 2 Pro and ZenFone AR to date). Owing to these pricey hardware requirements, the project recently shut down in March 2018 [35]. That project or later technology would promise a better performance than Smombie Guardian. This remains a consideration for our future work.

Finally, although we gave the participants a short 10-minute break after the first round of the study (walking along the path with Smombie Guardian turned off), our within-subjects study design might produce biased results, because the participants could have become familiar with the obstacles. Randomizing the order of the obstacles for each round would be more ideal. We will consider this for our future user studies.

## Conclusions

In this study, in order to remedy the lack of situational awareness in smartphone users as they walk outdoors, we introduced a smartphone app called Smombie Guardian, designed to redirect users' attention to their surroundings to prevent collisions. The results of our field test study involving 74 human subjects demonstrated the feasibility of the app with respect to its effectiveness, usefulness, and unobtrusiveness. We envision that such an alerting service will be embedded in smartphones as a native feature in the near future, and that the guidelines of our study will offer insights for such alert services.
